# Supplementary material for: Body Mass Index and Overall Survival of Patients with Newly Diagnosed Multiple Myeloma
Source: Cancers (Basel). 2022 Oct 29;14(21):5331. doi: 10.3390/cancers14215331 (PMC9657446; doi:10.3390/cancers14215331)
Supplement: Supplementary file 1 [file cancers-14-05331-s001.zip › cancers-1956720-supplementary.pdf]

Supplementary Table S1. Selected prognostic factors of multiple myeloma by body mass index (BMI) status at the time of diagnosis in the UChicago Multiple Myeloma (MM) Epidemiology Study, 2010-2019

	BMI status at diagnosis[a]			P value of $\chi 2^b$
	Normal[a]	Overweight	Obese	
	Number (%)			
**Age at diagnosis (years)**				
<60	56 (37.84)	76 (35.85)	71(38.59)	.47
60-69	53 (35.81)	91 (42.92)	78 (42.39)	
>=70	39 (26.35)	45 (21.23)	35 (19.02)	
**International staging system (ISS)**				
1	65 (61.90)	104 (67.53)	92 (63.89)	.56
2	29 (27.62)	40 (25.97)	35 (24.31)	
3	11 (10.48)	10 (6.49)	17 (11.81)	
**Estimated glomerular filtration rate (eGFR) (mL/min)**				
>=60	95 (67.86)	140 (67.96)	128 (70.33)	.85
<60	45 (32.14)	66 (32.04)	54 (29.67)	
**Serum free light chains**				
Low [<0.26]	36 (25.90)	43 (21.29)	33 (18.23)	.50
Normal [0.26-1.65]	37 (26.62)	51 (25.25)	53 (29.28)	
High [>1.65]	66 (47.48)	108 (53.47)	95 (52.49)	
**Elevated lactate dehydrogenase (LDH) levels (U/L)**				
Normal [< 240]	90 (78.95)	131 (75.29)	105 (67.31)	.08
Elevated [>= 240]	24 (21.05)	43 (24.71)	51 (32.69)	
**Number of high-risk cytogenetic abnormalities[c]**				
0	29 (60.42)	46 (65.71)	40 (72.73)	.41
1+	19 (39.58)	24 (34.29)	15 27.27)	

a. BMI was calculated as weight in kilogram (kg) divided by the square of height in meters (m^2) [weight (kg) / height $(m)^2$], using weight and height measured at the time of diagnosis. BMI status was defined as normal weight (BMI 18.5-24.9, reference), overweight (BMI 25-29.9), and obese (BMI ≥30.0).
b. P values for the Pearson's chi-squared statistic
c. High-risk MM-associated cytogenetic abnormalities include t(4;14), t(14;16), t(14;20), del(17/17p), and gain(1q) defined by the International Myeloma Working Group.

Supplementary Table S2. Multivariable-adjusted HRs for overall survival of multiple myeloma associated with body mass index (BMI) at time of diagnosis by sex and race/ethnicity in the UChicago Multiple Myeloma Epidemiology Study, 2010-2019[a]

	**White female**	**Black female**	**White male**	**Black male**
	HR (95% CI)[a]			
BMI status[a]	**N=129**	**N= 86**	**N=225**	**N=55**
Normal	1.0 (referent)	1.0 (referent)	1.0 (referent)	1.0 (referent)
Overweight	0.97 (0.44, 2.13)	0.35 (0.15, 0.79)	1.23 (0.66, 2.28)	1.42 (0.42, 4.83)
Obese	0.44 (0.19, 1.05)	0.19 (0.08, 0.46)	1.62 (0.85, 3.10)	4.39 (1.09, 17.70)

HRs: hazard ratios; CI: confidence interval;

a. HRs are based on models that include age (<60, 60-69, ≥70), sex (male/female), race (white/black), international staging system (1/2/3), elevated lactate dehydrogenase levels (normal/elevated), and estimated glomerular filtration rate (>=60/<60).

b. BMI was calculated as weight in kilogram (kg) divided by the square of height in meters (m^2) [weight (kg) / height $(m)^2$], using weight and height measured at the time of diagnosis. BMI status was defined as normal weight (BMI 18.5-24.9, reference), overweight (BMI 25-29.9), and obese (BMI ≥30.0).

Supplementary Figure S1. Overall survival curves by body mass index status at the time of diagnosis in the entire cohort and stratified by sex and race in the UChicago Multiple Myeloma (MM) Epidemiology Study, 2010-2019

(a). Overall

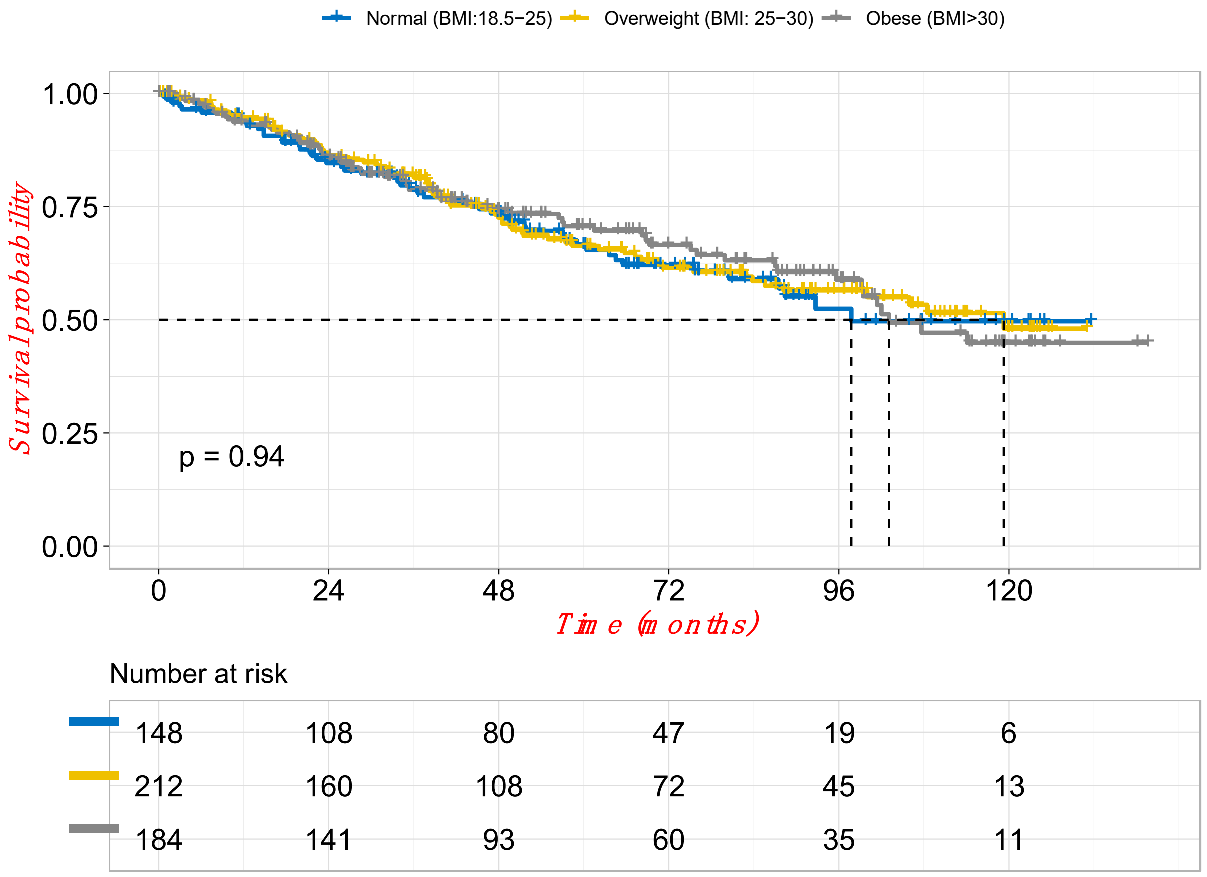

(b). Females

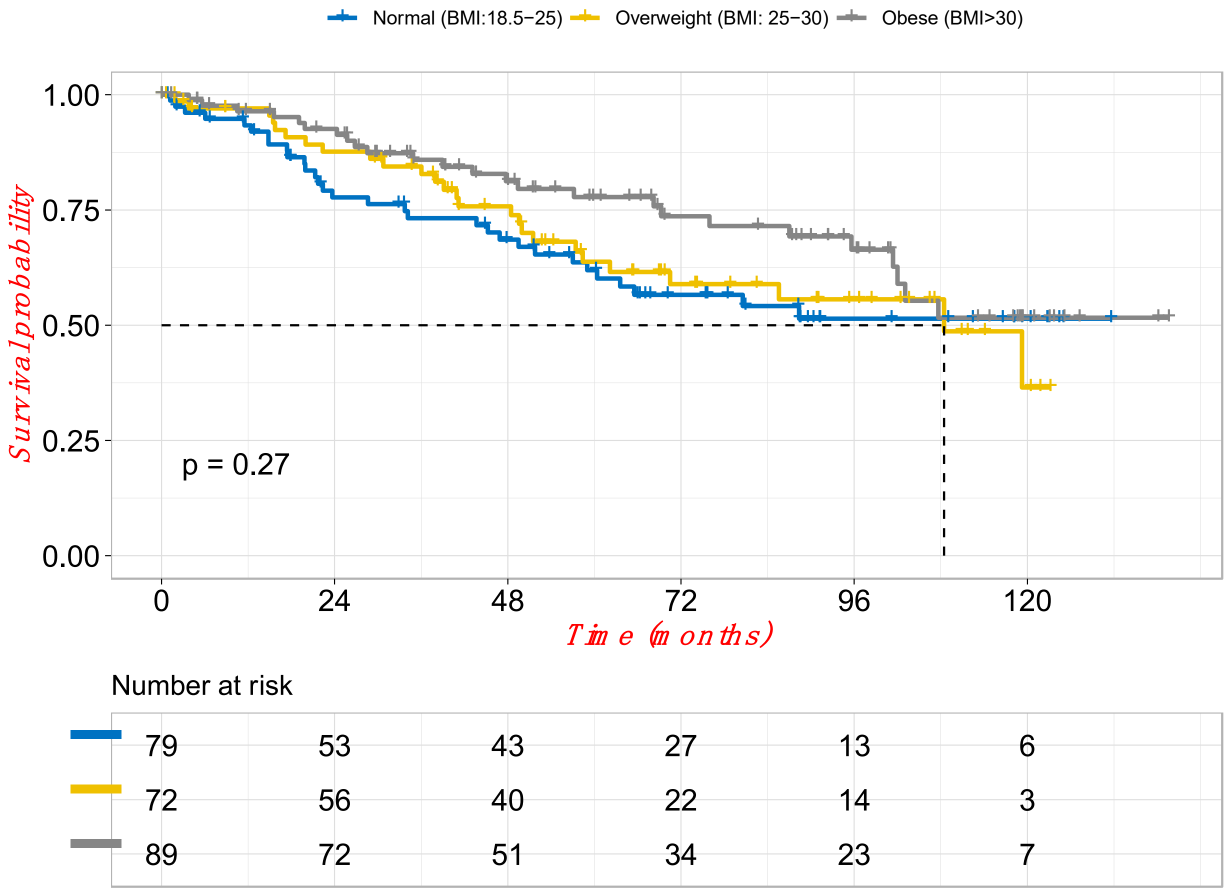
Normal (BMI:18.5−25)
Overweight (BMI: 25−30)
Obese (BMI>30)
1.00
0.75
0.50
0.25
0.00
Survival probability
p = 0.27
0
24
48
72
96
120
Time (months)
Number at risk
79 53 43 27 13 6
72 56 40 22 14 3
89 72 51 34 23 7

(c). Males

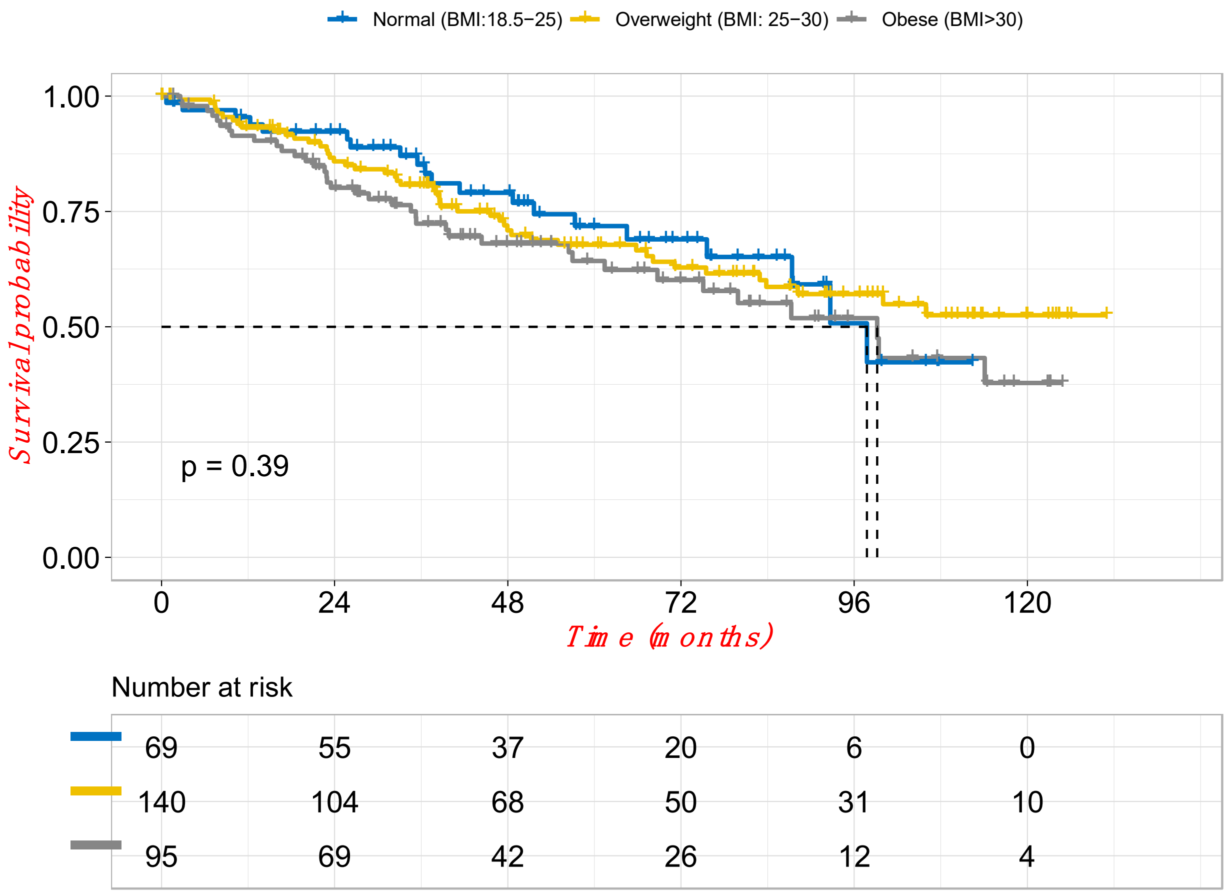

Normal (BMI:18.5−25)
Overweight (BMI: 25−30)
Obese (BMI>30)
1.00
0.75
0.50
0.25
0.00
Survival probability
p = 0.39
0
24
48
72
96
120
Time (months)
Number at risk
69 55 37 20 6 0
140 104 68 50 31 10
95 69 42 26 12 4

(d). Blacks

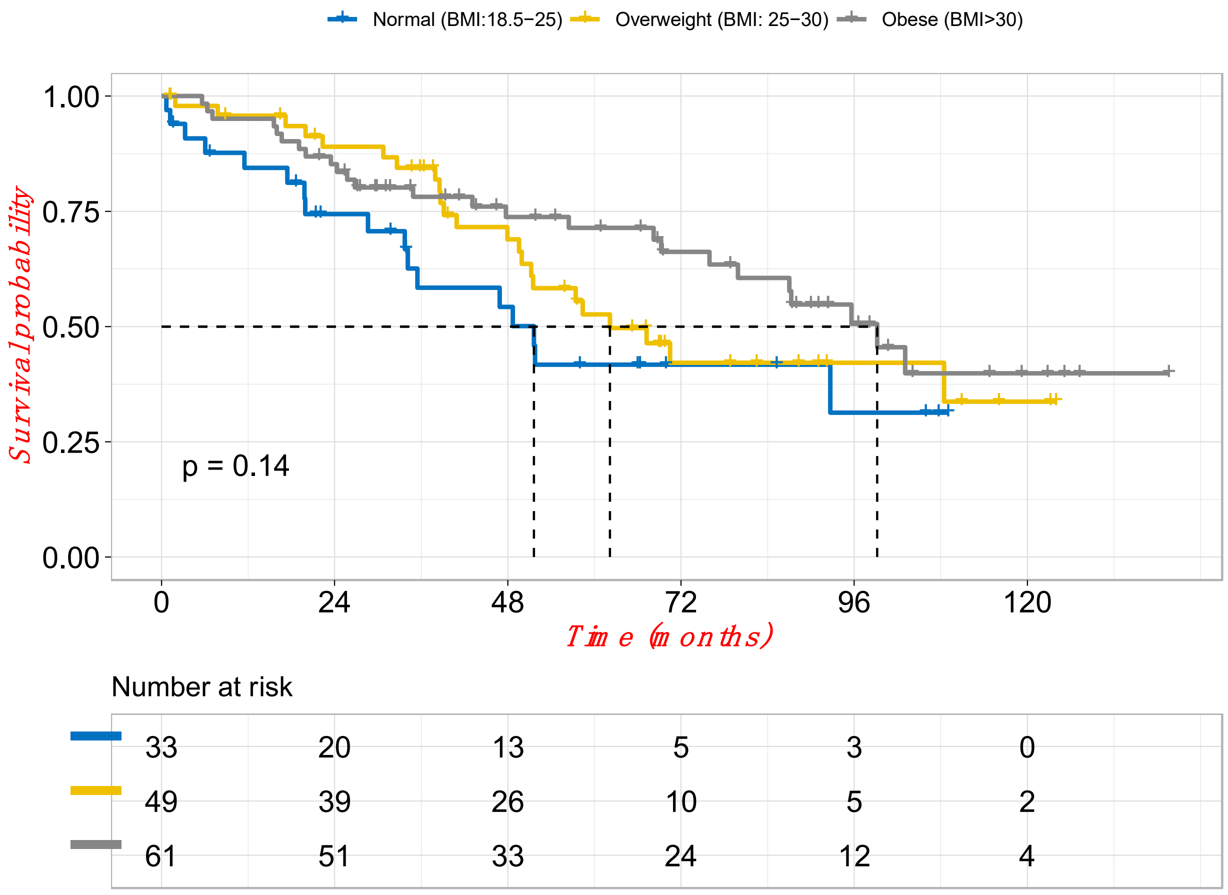
Normal (BMI:18.5−25)
Overweight (BMI: 25−30)
Obese (BMI>30)
Survival probability
1.00
0.75
0.50
0.25
0.00
p = 0.14
0
24
48
72
96
120
Time (months)
Number at risk
33 20 13 5 3 0
49 39 26 10 5 2
61 51 33 24 12 4

(e). Whites

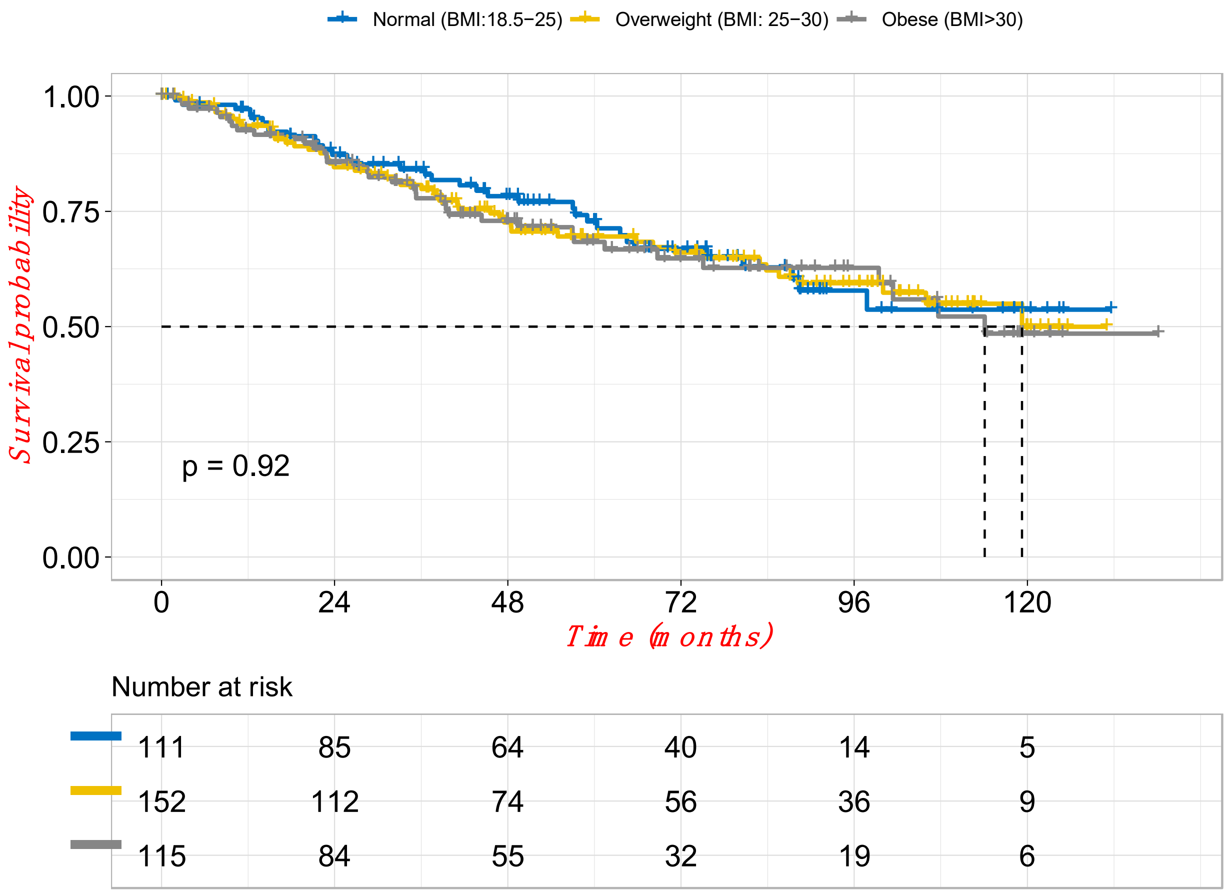
Normal (BMI:18.5−25)
Overweight (BMI: 25−30)
Obese (BMI>30)
1.00
0.75
0.50
0.25
0.00
Survival probability
p = 0.92
0
24
48
72
96
120
Time (months)
Number at risk
111 85 64 40 14 5
152 112 74 56 36 9
115 84 55 32 19 6
